# Testosterone Pellets in Women: Revisiting Safety and Clinical Outcomes

**DOI:** 10.7759/cureus.94442

**Published:** 2025-10-13

**Authors:** Diogo Pinto da Costa Viana, Leonardo Jacobsen, Luiz Henrique Gabriel, Eline Lobo de Souza Correia, Daniela da Maia Fernandes, Maria Luiza Nagel, Ana Comin

**Affiliations:** 1 Department of Gynecology, Paulista School of Medicine, Federal University of São Paulo, São Paulo, BRA; 2 Department of Gynecology, Brazilian Society for Research and Teaching in Medicine, Florianópolis, BRA; 3 Department of Gynecology, Brazilian Society of Obesity Medicine, Florianópolis, BRA; 4 Department of Gynecology, Brazilian Society for Research and Teaching in Medicine, Florianopólis, BRA; 5 Department of Gynecology, Brazilian Society for Research and Teaching in Medicine, Florianopolis, BRA

**Keywords:** androgens, bone density, cardiovascular health, cardiovascular safety, drug implants, hypoactive sexual desire disorder (hsdd), neoplasms, physiological sexual dysfunctions, postmenopause, testosterone

## Abstract

Testosterone is the most abundant biologically active gonadal steroid in women, yet its therapeutic role remains controversial. Current guidelines restrict its use to hypoactive sexual desire disorder (HSDD) with transdermal formulations, while subcutaneous pellets are used off-label in clinical practice despite unresolved concerns about pharmacokinetics, efficacy, and safety. The objective of this study is to critically evaluate the pharmacokinetics, clinical efficacy, and safety of subcutaneous testosterone pellets in peri- and postmenopausal women. A structured narrative review was conducted using PubMed/MEDLINE, covering studies published from January 1980 to August 2025. Other databases were not systematically searched. Search terms included “testosterone”, “pellets”, “implants”, “subcutaneous”, “women”, and “menopause”, combined with Boolean operators. Eligibility criteria comprised original pharmacokinetic or clinical studies in women, defined by STRAW+10 when available, reporting outcomes on pharmacokinetics, efficacy, or safety. Narrative synthesis was chosen due to heterogeneity in design, dosing, comparators, and outcome definitions. From 455 records, 38 studies were included. Pellets provided sustained release over four to six months with supraphysiologic early peaks (>100-250 ng/dL) and wide interindividual variability. The only randomized controlled trial showed improved sexual activity, orgasm, and satisfaction at 24 weeks with testosterone plus estradiol implants. Observational cohorts reported improvements in sexual function (Female Sexual Function Index, Female Sexual Distress Scale-Revised, and satisfying sexual events), mood, energy, and bone density, but findings are limited by non-randomized designs, lack of blinding, and conflicts of interest. Safety data, dominated by practice-based registries, indicated mild androgenic events (acne and hair growth) and rare transient voice changes; signals of reduced breast cancer incidence were reported but derive from cohorts without adequate adjustment for confounders, precluding causal inference. Data on cardiovascular, metabolic, and endometrial outcomes remain sparse and inconsistent. Testosterone pellets provide long-term delivery but at the cost of supraphysiologic peaks, dosing variability, and reliance on observational evidence. Reported benefits for sexual function and well-being are hypothesis-generating, while safety cannot be confirmed. Until adequately powered randomized trials with standardized formulations are conducted, pellet use should remain individualized, off-label, and accompanied by structured monitoring rather than routine adoption.

## Introduction and background

The pursuit of improved quality of life and health optimization during perimenopause and postmenopause has renewed interest in steroid hormones beyond estrogen. Testosterone, although traditionally labeled a “male hormone,” is the most abundant biologically active gonadal steroid across a woman’s lifespan and circulates at higher concentrations than estradiol [1]. Historically, however, it has been overlooked in women’s health despite its recognized role in sexual function, metabolic regulation, musculoskeletal health, and psychological well-being [2]. Androgen production declines gradually from the third decade of life, independently of menopause, and relative androgen insufficiency has been associated with reduced vitality, mood changes, unfavorable body composition, and diminished sexual desire [3,4].

Current international guidelines restrict therapeutic testosterone use in women to the treatment of hypoactive sexual desire disorder (HSDD) in postmenopausal women, based primarily on randomized controlled trials (RCTs) of transdermal formulations [4]. The evidence hierarchy is clear: most data derive from transdermal RCTs, only one small RCT has evaluated subcutaneous pellets, and the majority of the remaining evidence comes from observational cohorts. Nonetheless, professional societies such as the British Menopause Society (BMS) acknowledge that, in practice, testosterone therapy is sometimes considered more broadly, including the use of subcutaneous implants (pellets) when conventional hormone replacement therapy proves insufficient [5].

Pellets are small compressed crystalline implants inserted subcutaneously, designed to release testosterone gradually over several months. Their pharmacokinetic profile differs from transdermal formulations, as they typically produce an early supraphysiologic peak followed by a sustained plateau [6-9]. The definition of the “physiologic range” in women varies by assay type (liquid chromatography-tandem mass spectrometry (LC-MS/MS) vs. immunoassay), age, and whether total or free testosterone is measured. In postmenopausal women, total testosterone typically ranges from approximately 10 to 55 ng/dL by LC-MS/MS, and concentrations above this range are generally considered supraphysiologic, although exact cutoffs vary by assay and age. These nuances are critical for interpreting studies and for determining whether serum levels achieved with pellets fall within or above the expected female range.

Despite regulatory restrictions, the clinical use of testosterone pellets has expanded worldwide. In the United States, for example, compounded hormonal pellets account for an estimated 10-15% of testosterone prescriptions for women, reflecting both patient demand for convenience and clinician preference for sustained-release formulations. This widespread off-label use raises important clinical and regulatory concerns, given the absence of standardized manufacturing, dose consistency, and pharmacovigilance. Variability in compounding practices contributes to heterogeneous release kinetics and complicates safety interpretation.

Another source of controversy is the disconnect between guideline-directed targets and patient-reported outcomes. Clinical reports describe symptom improvement at serum concentrations above conventional laboratory thresholds, while regulators emphasize biochemical safety margins and physiologic dosing. Much of the available evidence originates from a limited number of research groups and practice-based registries, which increases the risk of bias and limits generalizability. These factors highlight the need for cautious interpretation, transparent reporting, and better alignment between clinical outcomes, laboratory targets, and regulatory standards.

Recent publications further illustrate these tensions. A 2025 review in the Revista da Associação Médica Brasileira critically discussed hormonal pellets but has been questioned for major methodological and ethical limitations. Although it claimed to apply GRADE methodology, criteria were not rigorously followed, and interpretation favored pellet use without adequate acknowledgment of evidence gaps. Importantly, both the authors and at least one peer reviewer disclosed or were later associated with conflicts of interest, raising concerns about bias in framing conclusions [10]. Conversely, a 2025 cohort study reported favorable biochemical profiles and symptom improvement with repeated pellet use for low libido, though its uncontrolled design and lack of comparators limit causal inference [11]. At the same time, safety concerns have been raised by case reports, including psychiatric manifestations temporally associated with pellet therapy, underscoring the importance of individualized monitoring and long-term safety studies [12]. Together, these heterogeneous reports highlight both the clinical enthusiasm for pellets and the persistent uncertainty regarding their regulation, safety, and reproducibility.

To address these issues, we conducted a structured narrative review to critically appraise the pharmacokinetics, clinical efficacy, safety, and practical application of subcutaneous testosterone pellets in women. The review integrates evidence from randomized and observational studies, with careful attention to methodological heterogeneity and the predominance of observational designs, and explicitly acknowledges the uncertainties that constrain definitive conclusions.

## Review

Materials and methods

A structured narrative review was conducted in accordance with the Scale for the Assessment of Narrative Review Articles (SANRA) guidelines. A comprehensive literature search was performed in PubMed/MEDLINE, covering studies published between January 1980 and August 15, 2025 (last search date). Both controlled vocabulary (Medical Subject Headings (MeSH)) and free-text terms were combined using Boolean operators. The search was restricted to human studies with full-text availability. Only articles written in English or Portuguese were included. Additionally, the reference lists of the included studies and relevant reviews were manually screened to identify further eligible publications. A line-by-line search strategy was applied in PubMed, considering both MeSH terms and title/abstract fields. We included Portuguese-language publications to capture regionally relevant evidence, while acknowledging that this decision could introduce language and selection bias. However, no Portuguese articles ultimately met the inclusion criteria. Additionally, the reference lists of the included studies and relevant reviews were manually screened to identify further eligible publications. A line-by-line search strategy was applied in PubMed, considering both MeSH terms and title/abstract fields (Table 1). The PubMed/MEDLINE database was considered sufficiently comprehensive for the scope and objectives of this narrative.

**Table 1:** Search query results MeSH, Medical Subject Headings; tiab, title/abstract

Search	Query	Results
#1	Search: women[MeSH Terms] OR women[tiab] OR female[tiab] OR Menopause[MeSH Terms] OR menopausal[tiab] OR Postmenopause[MeSH Terms] OR post-menopausal[tiab] OR Perimenopause[MeSH Terms] OR perimenopausal[tiab]	2,250,346
#2	Search: Humans[Mesh] OR Humans[tiab]	23,166,463
#3	Search: "testosterone"[MeSH Terms] OR testosterone[tiab]	118,514
#4	Search: implant*[tiab] OR pellet*[tiab] OR "subcutaneous"[tiab]	723,166
#5	Search: 1980"[Date - Publication] : "2025"[Date - Publication]	33,679,579
#6	Search: #1 AND #2 AND #3 AND #4 AND #5	455

A total of 455 records were identified. After title and abstract screening, 348 were excluded. A total of 107 full-text articles were assessed, and 38 met the inclusion criteria and were described in the review. A formal flow diagram was not prepared, as the selection process did not fully adhere to the structure of a systematic review but maintained transparency and traceability through the detailed description of the search strategy and inclusion criteria.

We included original pharmacokinetic and clinical studies evaluating subcutaneous testosterone pellets in women, reporting outcomes related to pharmacokinetics, efficacy, or safety. The primary population consisted of peri- or postmenopausal women, with postmenopause defined according to the STRAW+10 or equivalent criteria (≥12 months of amenorrhea and follicle-stimulating hormone levels within the menopausal range), when available. Comparator groups (estradiol alone, other testosterone formulations, or no therapy) were included when reported.

Exclusion criteria were follow-up shorter than four weeks; studies limited to men; those focused exclusively on non-pellet formulations; abstracts without full text; conference proceedings; editorials, commentaries, or expert opinions without original data.

Titles and abstracts were screened for relevance, followed by full-text review. The assessment was conducted by a single reviewer, with secondary verification when required. From each eligible study, we extracted the study design, sample size, population characteristics, dosage, follow-up duration, outcomes, adverse events, and, when available, funding sources and conflicts of interest.

Due to heterogeneity in dosing regimens, outcome definitions, pharmacokinetic assays, comparators, and follow-up periods, a narrative synthesis approach was adopted. Studies were organized thematically (pharmacokinetics, efficacy, and safety) and synthesized narratively. The high degree of variability in study design and reporting precluded quantitative pooling. Missing or unclear data were reported transparently, and potentially duplicated datasets were excluded when identified.

Methodological limitations, potential sources of bias, and conflicts of interest were qualitatively appraised for each study. The provenance and strength of the available evidence were explicitly described to support interpretation. Indirect evidence from male or transgender populations was treated as hypothesis-generating only, without extrapolation to women.

Results

Pharmacokinetics

The pharmacokinetics of subcutaneous testosterone implants are a central determinant of their clinical applicability and, simultaneously, the main source of controversy. Unlike formulations delivered through membranes or vehicles, pellets are compacted cylinders of crystalline testosterone manufactured by fusion, releasing the hormone through gradual dissolution and surface erosion. This process produces a profile characterized by an initial rise, often exceeding female physiologic ranges, followed by a plateau phase and a slow decline over several months [13].

Early studies first described this profile. Thom et al. (1981) [14], in oophorectomized women, reported that 100 mg implants increased total serum testosterone from 29 ng/dL to 144 ng/dL within approximately four weeks, with levels sustained for up to eight months. Kapetanakis et al. (1982) [15] found similar results, with 75 mg implants raising mean levels to 250 ng/dL and declining to baseline within four to five months. These small, heterogeneous trials established the general pattern: long duration of release coupled with transient peaks that frequently exceed female reference ranges (often cited as 15-70 ng/dL for total testosterone in postmenopausal women, depending on assay and age [16]). Reference intervals are assay- and age-dependent; LC-MS/MS-based ranges for postmenopausal women are typically narrower/lower (≈10-55 ng/dL) than those derived from older immunoassays.

Later work confirmed these patterns with more precise assays. For example, Buckler et al. (1998) [9] reported that a 100 mg pellet produced mean levels of 257 ng/dL at approximately one month, declining to 84 ng/dL at approximately six months. That study used immunoassay rather than LC-MS/MS; subsequent studies employing LC-MS/MS in women have likewise demonstrated early supraphysiologic peaks followed by prolonged release. These supraphysiologic excursions defined here against total testosterone reference ranges appropriate for postmenopausal women and the specific assay used are a basis for guideline caution, since current recommendations prioritize maintaining concentrations within female physiologic limits.

Although non-inferiority data in hypogonadal men suggest comparable pharmacokinetics between compounded pellets and the FDA-approved product Testopel [15], this constitutes indirect evidence and should not be extrapolated to women. Compounded implants used in clinical practice may vary in purity, sterility, potency, and release kinetics, and they are not subject to consistent regulatory oversight. This lack of standardization contributes to pharmacokinetic inconsistency and remains a central concern in safety debates.

Some investigators have proposed that symptom improvement may occur at concentrations above standard laboratory cutoffs. Glaser et al. (2013) [13], using a weight-based dosing strategy (~2 mg/kg), reported a mean total testosterone of 299 ng/dL at approximately four weeks post-insertion, with concentrations at reinsertion still averaging 171 ng/dL, approximately three to four times higher than typical LC-MS/MS-based upper limits (≈10-55 ng/dL), depending on age and laboratory. While such findings highlight the “pharmacokinetic paradox” between laboratory thresholds and reported benefits, they remain hypothesis-generating and must be interpreted with caution. Higher exposures are associated with potential risks, including androgenic adverse effects (acne, hirsutism, alopecia, voice change, and clitoromegaly), erythrocytosis, and metabolic changes (lipids and liver enzymes), which necessitate structured monitoring [16].

Comparisons with other formulations contextualize this debate. Oral testosterone undecanoate is associated with erratic absorption, high peaks, and adverse lipid effects [4,8,16]. Intramuscular injections produce sharp fluctuations leading to symptomatic variability [17,18]. Transdermal preparations provide more stable release but require daily application, carry risks of transfer, and may still induce supraphysiologic levels at higher doses [19,20]. Pellets, in contrast, provide long-term exposure at the cost of early supraphysiologic peaks and limited reversibility once implanted.

Another important aspect is interindividual variability. Even at standardized doses, Glaser et al. (2013) [13] reported up to fourfold differences in total serum testosterone. Variability is influenced by body mass index, vascularization, metabolism, and androgen receptor sensitivity, as well as by pellet composition/dose, insertion site, and assay timing (including diurnal variation) [3]. These factors complicate the definition of a universal therapeutic range, since women with very different serum concentrations may show similar symptom responses and tolerability.

**Figure 1:**
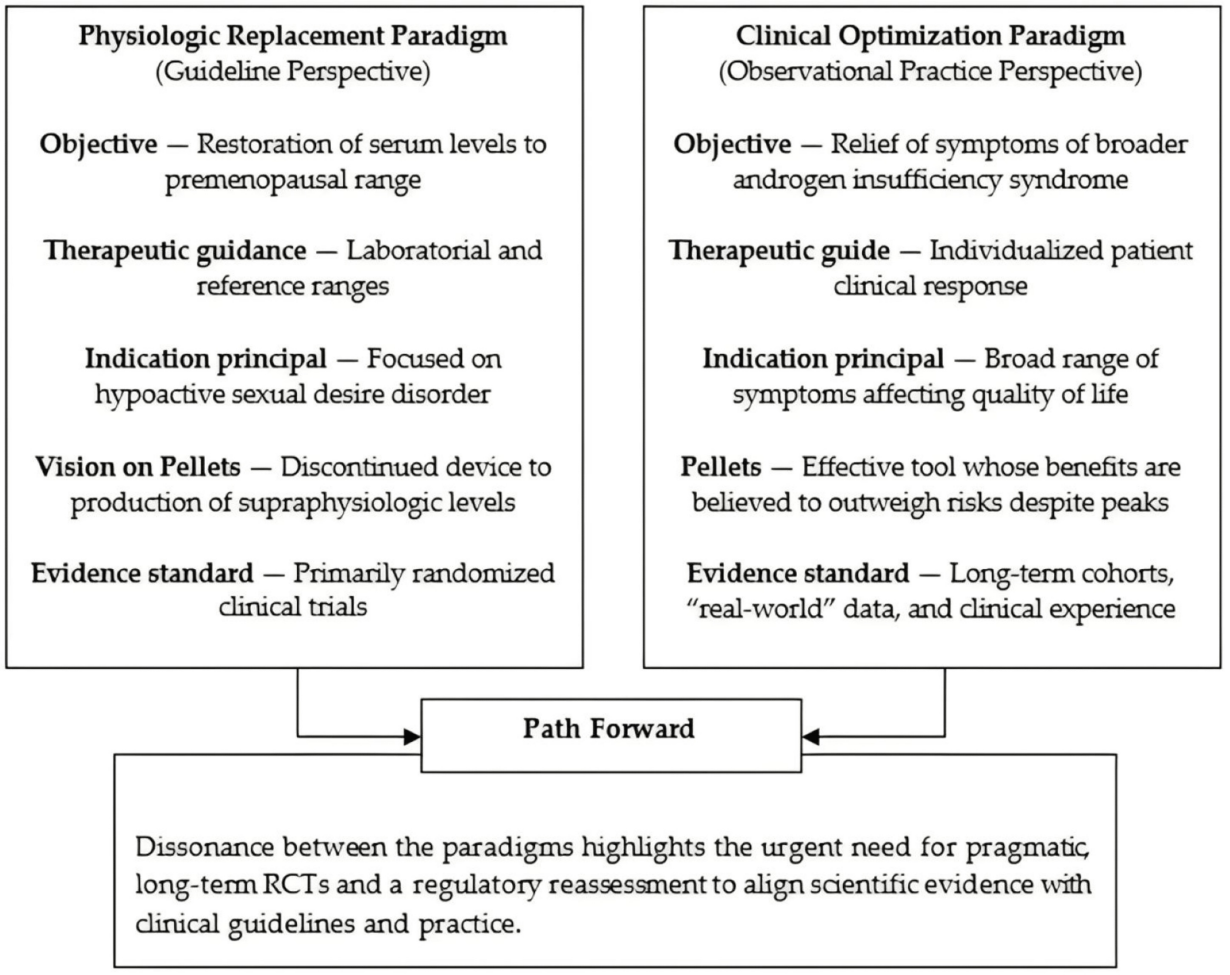
“Pharmacokinetic paradox” of testosterone pellets Conceptual representation of the serum testosterone trajectory after pellet implantation, showing an initial supraphysiologic peak followed by a plateau and gradual decline. The figure illustrates the discrepancy between laboratory-defined physiologic ranges (LC-MS/MS upper limit of approximately 55 ng/dL for postmenopausal women) and the symptomatic relief often reported at higher levels. LC-MS/MS, liquid chromatography-tandem mass spectrometry; RCT, randomized controlled trial

Taken together, available data confirm that pellets achieve long-term release but at the expense of early supraphysiologic peaks and wide interindividual variability. This duality explains both their clinical appeal and the reluctance of guidelines to endorse them. The debate illustrates the gap between a laboratory-centered paradigm and a symptom-centered approach, but conclusions must remain tentative given the small number of trials, concentration of data in a few research groups, and absence of adequately powered comparative studies. Figure 1 illustrates median serum trajectories with interquartile ranges from representative female cohorts (assay specified per dataset), highlighting the characteristic early peaks followed by prolonged plateaus; underlying timepoints and sources are listed in Table 2.

**Table 2:** Pharmacokinetic characteristics of different testosterone formulations in women TU, testosterone undecanoate; ULN, upper limit of normal

Formulation	Release profile	Peak serum levels	Duration of action	Advantages	Limitations
Subcutaneous pellets	Gradual dissolution and erosion [21,22]	Initial supraphysiologic peak (2-4× ULN) [21,22]	Three to six months [14,21]	Convenience, sustained release [14,22]	Cannot be promptly discontinued, interindividual variability [15,22]
Oral (TU)	Erratic absorption, first-pass hepatic metabolism [15]	Peaks up to 10× physiologic [17]	Hours [17]	Oral route [15]	Hepatotoxicity, lipid adverse effects, unpredictable [17]
Intramuscular (cypionate/enanthate)	Depot, rapid rise then sharp decline [15]	Supraphysiologic immediately post-injection [17]	One to three weeks [17]	Widely available [15]	Fluctuations, mood swings, injection-site discomfort [17]
Transdermal (gel/patch)	Continuous, steady [15]	Within physiologic range if adherence is maintained [17]	Daily [17]	Stable, noninvasive [17]	Variable absorption, transfer risk, adherence burden [17]

Testosterone remains the androgen with the strongest evidence for efficacy in female sexual health, with treatment of HSDD as the only indication formally recognized by international guidelines. The meta-analysis by Islam et al. (2019) [23], which informed the Global Consensus, demonstrated that testosterone therapy, particularly via transdermal formulations, significantly improved desire, arousal, orgasm, and frequency of satisfying sexual events (SSEs). These outcomes were assessed using validated endpoints such as the Female Sexual Function Index (FSFI), the Female Sexual Distress Scale-Revised (FSDS-R), and SSEs, typically at 12-24 weeks.

For subcutaneous implants, early studies reported associations with increased libido [22,23]. The RCT by Davis et al. (1995) [24] remains the only controlled trial specific to pellets, showing that the addition of 50 mg of testosterone to estradiol implants improved sexual activity, satisfaction, pleasure, and orgasm compared with estradiol alone after 24 weeks. These findings have been supplemented by observational cohorts [24], but interpretation must remain cautious given their non-randomized design, lack of blinding, and limited control of confounders.

In addition to these earlier studies, newer long-term observational data have examined pellet therapy in women with low libido. A 2025 cohort study reported favorable biochemical profiles and symptomatic improvement with repeated insertions, but the uncontrolled design and absence of a comparator arm limit causal inference [11].

Beyond HSDD, a variety of somatic and psychological effects have been hypothesized. Early reports described improvements in fatigue and concentration [25] and mood-related symptoms when testosterone was combined with estradiol implants [26]. However, case-based evidence has also described adverse neuropsychiatric manifestations, including a recent report of mood destabilization in a postmenopausal user of pellets [12], reinforcing the need for careful monitoring. Observational cohorts such as Glaser et al. (2011) [25] have reported improvements in Menopause Rating Scale (MRS) domains, such as vasomotor, musculoskeletal, sleep, mood, and energy, but these results derive from uncontrolled settings and may reflect expectancy effects.

Pilot studies have also explored other symptomatic domains, such as migraine reduction. Reports of high proportions of women experiencing fewer attacks (e.g., up to 74%) come from uncontrolled or small exploratory cohorts [27] and therefore require confirmation in adequately powered randomized trials before being interpreted as evidence of efficacy.

Changes in body composition have been reported in both controlled and uncontrolled settings. In the Davis et al. trial (1995) [24], women receiving estradiol plus testosterone had increases in lean body mass compared with estradiol alone after 24 weeks. Practice-based registries, such as those by Donovitz (2022) [8], have described improvements in muscle strength, vitality, and body composition, but these findings arise from retrospective observational data with potential conflicts of interest, heterogeneous dosing, and no standardized outcome assessment. As such, they should be considered exploratory.

Skeletal effects have also been investigated. Testosterone may influence bone through direct anabolic effects on osteoblasts and aromatization to estradiol [16]. In the Davis et al. trial (1995) [24], greater increases in bone mineral density were observed at the whole body, lumbar spine, and trochanteric sites compared with estradiol alone over six months. The 2019 Global Consensus [5] deemed evidence insufficient overall, mainly because most studies used transdermal formulations and lacked long-term follow-up. Findings from pellet cohorts offer hypothesis-generating signals but remain limited by study design and the absence of randomized replication.

A consolidated summary of reported outcomes, including sexual function, psychological well-being, body composition, and bone health, is presented in Table 2. While symptomatic improvements are frequently described, the predominance of observational and practice-based data limits the ability to infer causality or generalize results.

Clinical Efficacy of Testosterone Pellets in Women

Testosterone remains the androgen with the strongest evidence for efficacy in female sexual health, with the treatment of HSDD as the only indication formally recognized by international guidelines. The meta-analysis by Islam et al. (2019) [23], which informed the Global Consensus, demonstrated that testosterone therapy, particularly via the transdermal route, significantly improved multiple domains of sexual function, including desire, pleasure, arousal, orgasm, and frequency of SSEs. With respect to subcutaneous implants, early studies had already associated their use with improved libido [14,28]. The RCT by Davis et al. (1995) [24] remains the most robust controlled evidence for pellets, showing that the addition of 50 mg of testosterone to estradiol implants significantly enhanced sexual activity, satisfaction, pleasure, and orgasm compared with estradiol alone. These findings have been complemented by observational cohorts, though interpretation must remain cautious given their non-randomized and practice-based design [25].

Beyond HSDD, a range of somatic and psychological benefits has been proposed. Early reports described improvements in fatigue and concentration [26], as well as reductions in anxiety and depression when testosterone was combined with estradiol implants [29]. More recent cohorts, such as Glaser et al. (2011) [25], documented improvements across all domains of the MRS, including vasomotor, musculoskeletal, sleep, mood, and energy-related symptoms. A pilot study from the same group also reported a reduction in migraine frequency, with 74% of women remaining attack-free during treatment [27]. These findings, while encouraging, remain hypothesis-generating and require confirmation through RCTs.

Effects on body composition have also been described. Davis et al. (1995) [24] reported an increase in lean body mass among women receiving estradiol plus testosterone compared with estradiol alone, suggesting anabolic effects. Observational practice-based cohorts, including those by Donovitz (2022) [8], have further suggested improvements in muscle strength and vitality, but these data derive from retrospective registries with potential conflicts of interest and should be considered low-level evidence, hypothesis-generating rather than confirmatory.

Skeletal health is another area of interest. Testosterone exerts direct anabolic actions on osteoblasts and serves as a precursor for estradiol; both mechanisms are important for bone maintenance [16]. In the trial by Davis et al. (1995) [24], women receiving estradiol plus testosterone implants demonstrated significantly greater increases in bone mineral density of the whole body, lumbar spine, and trochanteric region compared with estradiol alone, with effects detectable within six months. Although the 2019 Global Consensus [5] concluded that the overall evidence was insufficient, primarily due to reliance on transdermal studies, findings from pellet cohorts provide additional support for a possible role in bone health.

A consolidated summary of clinical outcomes reported with pellet therapy, including sexual function, psychological well-being, body composition, and bone health, is presented in Table 3. This table highlights both the consistency of reported symptomatic improvements and the limitations imposed by reliance on observational and practice-based cohorts.

**Table 3:** Clinical outcomes reported with subcutaneous testosterone pellets in women, including effects on sexual function, psychological well-being, body composition, and bone health ^*^ Data from practice-based registries with potential conflicts of interest; considered low-level and hypothesis-generating rather than confirmatory. BMD, bone mineral density; RCT, randomized controlled trial

Outcome domain	Key findings	Study type	Notes
Sexual function	Improved desire, orgasm, and satisfaction [7,23,28]	RCT, cohorts	Only one RCT; observational cohorts dominate
Psychological well-being	Reduced anxiety/depression, improved energy [28]	Cohorts	Non-randomized, patient-reported
Body composition	↑ Lean mass, muscle strength [7,23]	RCT, registries	Registry data with COI^*^
Bone health	↑ BMD lumbar spine/trochanter [23]	RCT	Effect independent of estradiol suggested

Safety of Testosterone Pellet Therapy

The most common adverse effects are androgenic in nature. The meta-analysis by Islam et al. (2019) [23], largely based on transdermal formulations, reported increased risks of acne (RR 1.46) and hair growth (RR 1.69) compared with placebo, without higher discontinuation rates. Severe effects such as clitoromegaly, alopecia, or voice deepening were not observed. In pellet-specific cohorts, findings are broadly similar. Glaser et al. (2013) [13] reported mild-to-moderate facial hair in 85.7% of women, moderate acne in 11.2%, transient hoarseness in 1%, and no clitoromegaly. Hernandez et al. (2025) [11], using lower doses (75-100 mg), described a lower incidence, with only 5.3% reporting new facial hair. These findings suggest that androgenic effects are generally mild, though their prevalence highlights the need for proactive monitoring and transparent patient counseling.

Androgenic and other effects: The most frequent adverse effects of testosterone therapy in women are androgenic. In the meta-analysis by Islam et al. (2019) [23], which primarily evaluated RCTs of physiologic transdermal dosing over 12-24 weeks, acne was more common with therapy (RR 1.46; 95% CI 1.11-1.92), as was increased hair growth (RR 1.69; 95% CI 1.33-2.14) versus placebo. Baseline event rates in the included trials were low (generally <5%), so the absolute risk differences correspond to approximately three to five additional cases per 100 treated women over 12-24 weeks. Importantly, therapy was not associated with higher rates of alopecia, voice deepening, or clitoromegaly, and discontinuation rates did not differ from placebo.

When specifically considering pellets, which often achieve pharmacologic exposures with early supraphysiologic peaks, the available data come almost exclusively from observational cohorts. In the prospective study by Glaser et al. (2013) [13], androgenic adverse events were actively collected via structured questionnaires: 85.7% reported mild-moderate facial hair growth, 11.2% moderate acne, 1% transient hoarseness, and no clitoromegaly during a mean follow-up of 20 months. In contrast, Hernandez et al. (2025) [11], using lower doses (75-100 mg) and longer reinsertion intervals, reported 5.3% new-onset facial hair and 2.6% acne. These studies employed non-randomized, practice-based designs; ascertainment relied on patient self-report without standardized grading (e.g., Common Terminology Criteria for Adverse Events), which may under-ascertain mild/transient events and is underpowered to detect rare or irreversible outcomes (e.g., persistent voice change and clitoromegaly).

Comparisons to data from transgender men provide a mechanistic context for androgen-driven virilization, but direct extrapolation is inappropriate because of differences in physiology, dose targets, routes, baseline risk, monitoring, and concomitant therapies. In cis women, current evidence supports that pellet therapy increases the likelihood of mild androgenic effects, whereas the risk of severe or irreversible virilization appears low but cannot be excluded given limited sample sizes and follow-up.

Beyond androgenic outcomes, evidence on hematologic and metabolic safety with pellets remains sparse. Transdermal RCTs show small hematocrit increases but not frank erythrocytosis [13]. In pellet cohorts, modest hematocrit rises have been described, yet systematic reporting is uncommon. Data on lipids, liver enzymes, blood pressure, and glucose-insulin parameters are limited and inconsistent, with insufficient power to exclude adverse trends; these domains therefore remain a key evidence gap.

A potential dose-response has been observed: higher implant doses (>100-150 mg) and shorter reinsertion intervals are associated with more androgenic events and higher hematocrit [9,27]. On this basis, expert practice recommends structured monitoring: total testosterone (preferably by LC-MS/MS), hematocrit, and liver function tests at three to six months after insertion and at each reinsertion; defer reinsertion or consider explant if testosterone remains supraphysiologic, hematocrit exceeds 48-50%, or if significant androgenic adverse events occur. Practical constraints, including access to and cost of LC-MS/MS in low female ranges, turnaround times, and availability of standardized adverse event (AE) grading, may limit routine monitoring in some settings and should be addressed during shared decision-making.

Cardiovascular safety: Cardiovascular risk represents a central barrier to wider acceptance of pellet therapy. Observational studies have reported that lower endogenous testosterone in women is associated with higher rates of ischemic heart disease and all-cause mortality [16,28], but the clinical relevance of exogenous replacement remains uncertain. In the meta-analysis by Islam et al. (2019) [23], oral testosterone was associated with adverse changes in lipid profiles, whereas non-oral formulations, including transdermal and subcutaneous pellets, did not consistently demonstrate such effects. Earlier trials support this pattern: Davis et al. (1995) [24] found that adding a 50 mg testosterone pellet to estradiol preserved favorable lipid changes, while Burger et al. (1984) [26] reported no significant alterations despite supraphysiologic peaks. More recently, large-scale database and cohort analyses, including van Staa and Sprafka (2009) [30] and Hernandez et al. (2025) [11], found no excess risk of ischemic heart disease, stroke, erythrocytosis, or hypertension among pellet users, though statistical power to detect rare outcomes was limited.

Additional context comes from long-term studies in transgender men receiving supraphysiologic testosterone, which overall show no consistent increase in cardiovascular mortality compared with reference populations [30,31]. However, these findings should be regarded as indirect evidence, as baseline physiology, comorbidities, dosing regimens, and monitoring practices differ substantially from those in peri- and postmenopausal women.

The clinical use of subcutaneous testosterone pellets in women illustrates the persistent gap between guideline-based recommendations and real-world practice. Most international societies restrict testosterone therapy to postmenopausal women with HSDD and favor transdermal formulations [4,32]. A recent clinical overview reiterated this position, emphasizing that transdermal testosterone remains the preferred route, while other formulations, such as pellets, are occasionally used in practice but lack regulatory approval [21]. However, the BMS [5] acknowledges that testosterone may be considered in women with persistent symptoms unrelieved by conventional therapies and notes that subcutaneous implants (pellets) are occasionally used in UK practice as part of individualized strategies. Such acknowledgment underscores the ongoing clinical demand for alternatives beyond approved formulations.

This divergence is reflected in the so-called “pharmacokinetic paradox.” While laboratory-defined female reference ranges for total testosterone commonly cite upper limits of ~10-55 ng/dL depending on assay type, age band, and laboratory (with LC-MS/MS-based ranges typically narrower and lower than immunoassay-derived values), symptomatic benefit has been reported at concentrations well above these thresholds. Several studies describe therapeutic windows extending into ranges often classified as supraphysiologic [9]. Recent perspectives have also emphasized that testosterone is the most abundant biologically active gonadal steroid across a woman’s lifespan, yet it has historically been underrecognized in clinical medicine [2]. This discrepancy highlights the limitations of frameworks that prioritize biochemical targets, while clinical practice often emphasizes patient-reported outcomes.

Safety remains the most debated domain. Data from the Dayton prospective cohorts (n ≈ 1200 women, ~7000 person-years of pellet exposure) reported breast cancer incidence rates lower than age-standardized Surveillance, Epidemiology, and End Results (SEER) expectations, with hazard ratios ranging from 0.45 to 0.65 (95% CI 0.25-1.00) [10]. Retrospective registry analyses, such as those by Donovitz (2022) [8] and Donovitz and Cotten (2021) [33], have shown similar signals, but these derive from practice-based cohorts with industry/clinic affiliations and should be considered hypothesis-generating rather than confirmatory. Importantly, ascertainment methods, follow-up duration, and adjustment for confounders varied, limiting the reliability of causal inference. In addition, isolated case reports have described psychiatric manifestations associated with prolonged pellet therapy, underscoring the need for vigilance regarding psychological domains [12].

Cardiovascular outcomes show a comparable pattern. In a retrospective analysis of ~4000 pellet users followed for >10,000 person-years, Hernandez et al. (2025) [11] reported no excess of hypertension, ischemic heart disease, or thromboembolic events compared with population norms. However, effect estimates were imprecise (adjusted HRs ~1.0, 95% CI 0.7-1.5), and ascertainment relied on clinical coding rather than adjudicated endpoints. These data provide indirect reassurance but remain insufficient to establish safety equivalence with approved formulations. Contextual information from transgender men, who maintain supraphysiologic testosterone levels for decades without consistent increases in cardiovascular mortality [30,31], should be interpreted cautiously, given differences in dose targets, treatment regimens, baseline risk, and monitoring practices.

Conflict of interest and provenance remain critical considerations. Much of the pellet literature originates from a small number of research groups and practice-based registries with potential industry affiliations. These ties may introduce reporting bias and overestimation of benefit. Accordingly, conclusions regarding oncologic and cardiovascular safety must remain tentative.

From a clinical perspective, pellets may be considered in carefully selected women with persistent symptoms despite conventional therapy, but only under structured monitoring and shared decision-making. At a minimum, monitoring should include total testosterone (preferably LC-MS/MS), hematocrit, and liver function tests at baseline and three to six months after insertion, with repeat testing at each reinsertion. Re-implantation should be delayed if testosterone concentrations remain supraphysiologic, hematocrit exceeds 48-50%, or clinically significant androgenic adverse events occur.

Future research should address these gaps with pragmatic RCTs comparing pellets with approved testosterone formulations. Primary endpoints should include validated measures such as FSFI, FSDS-R, SSEs, and MRS domains, assessed at 12, 24, and 52 weeks. Safety core sets should cover androgenic adverse events, hematocrit, lipid profile, liver enzymes, blood pressure, and breast and endometrial outcomes. Standardization of pellet composition, dose, and reinsertion intervals, as well as uniform use of LC-MS/MS for assay precision, are essential to harmonize findings and strengthen the evidence base.

Endometrial safety: Evidence regarding the endometrium is limited but suggests an overall neutral or protective profile. Early histological reports described atrophic endometrium in women receiving estradiol plus testosterone implants, with no evidence of hyperplasia or malignancy during follow-up [34,35]. Subsequent observational series confirmed that endometrial safety outcomes were not adversely affected, although biopsy-verified long-term data remain sparse. The absence of large prospective cohorts with systematic endometrial surveillance limits definitive conclusions.

Breast safety: Observational cohorts such as Donovitz et al., Donovitz and Cotton, and Glaser et al. [1,33,36] reported lower-than-expected breast cancer incidence in pellet users, though these findings derive from practice-based registries with potential conflicts of interest and selection bias. Long-term retrospective analyses have described incidence rates below epidemiological expectations, but the lack of randomization, absence of standardized monitoring, and limited control of confounders preclude causal inference. These data should be interpreted as hypothesis-generating signals rather than proof of safety, and replication by independent groups is required to mitigate concerns of bias and conflicts of interest.

Indications and Dosing Rationale

The clinical application of subcutaneous testosterone pellets in women has historically focused on HSDD, the only indication formally endorsed by international consensus [5,23]. However, clinical practice demonstrates that women often present with a broader spectrum of symptoms, and the BMS [6] explicitly acknowledges that pellets are occasionally used in the UK as part of individualized management when conventional strategies fail. This reflects an ongoing tension between guideline conservatism and real-world therapeutic demand.

A key consideration is the relationship between pellet pharmacokinetics and endogenous physiology. In healthy premenopausal women, total daily testosterone production is estimated at 0.6-0.8 mg, with roughly equal ovarian and adrenal contributions [14,22]. Pharmacokinetic studies demonstrate that a 50 mg pellet releases approximately 0.3-0.4 mg/day, close to ovarian production, while 75-100 mg provides 0.6-0.8 mg/day, effectively mirroring the combined ovarian-adrenal output [13,22]. These data argue against the frequent perception that all pellet regimens are inherently supraphysiological. Instead, supraphysiologic exposure occurs primarily with higher doses (125-200 mg), which exceed 0.9-1.2 mg/day and therefore should be restricted to exceptional cases under rigorous monitoring, as summarized in Table 4.

**Table 4:** Estimated daily testosterone release from subcutaneous pellets at different doses, compared with endogenous ovarian and adrenal production Data illustrate the concept of physiologic equivalence, whereby 50 mg pellets approximate ovarian output, 75-100 mg reflect combined ovarian and adrenal production, and higher doses (>125 mg) exceed physiologic levels and should be reserved for selected cases under strict monitoring.

Pellet dose (mg)	Estimated release (mg/day)	Physiologic equivalence	Duration of effect
50 mg	Derivative (0.3-0.4) — linear extrapolation from 200 mg (1.3-1.5 mg/d ≈0.335 mg/d for 50 mg)	Not applicable	Four to six months [33,36]
75 mg	Direct (~0.6 mg/d per 75 mg pellet)	Not applicable	Four to six months [37]
100 mg	Derivative (≈0.65-0.75 mg/d) — proportional to 200 mg (1.3-1.5 mg/d)	Not applicable	Four to six months [33,36]
125-150 mg	Derivative (0.9-1.0 mg/d) — proportional to 200 mg	Not applicable	Four to six months [33,36]
200 mg	Direct (1.3-1.5 mg/d per 200 mg pellet)	Not applicable	Four to six months [33,36,37]

This comparison between physiologic production and pellet release highlights the principle of physiologic equivalence: dosing strategies should aim to approximate endogenous output rather than exceed it. Aligning therapy with natural physiology not only provides a rational framework for clinical decision-making but also counters the criticism that pellet therapy inevitably produces unsafe exposure levels. By embedding pharmacological rationale into clinical practice, physicians can better balance efficacy with safety and move toward standardized dosing strategies.

Clinical Application

The clinical application of subcutaneous testosterone pellets in women is supported primarily by their ability to provide sustained hormone release over several months, improving adherence compared with daily formulations. Glaser et al. (2013) [13] reported that with a weight-based regimen of approximately 2 mg/kg, mean serum testosterone levels reached 299 ng/dL four weeks after insertion and declined to 171 ng/dL at the time of symptom recurrence. Although these concentrations exceeded the physiologic range, they were associated with reported symptomatic improvement. From a practical standpoint, pellets are valued for their convenience and consistent symptom control, particularly in women with HSDD or in those experiencing persistent menopausal symptoms not adequately controlled by estrogen therapy.

Despite these advantages, the pharmacokinetic characteristics of pellets introduce relevant limitations. The unavoidable supraphysiologic peaks may provoke transient androgenic effects, and the inability to rapidly discontinue exposure in the event of adverse reactions is a significant clinical challenge. Therefore, careful patient selection and rigorous monitoring are essential. Follow-up should include symptom evaluation, periodic serum testosterone measurement, and surveillance of breast and endometrial health. Discontinuation must be considered in cases of persistently elevated levels, clinically significant side effects, or patient dissatisfaction.

Evidence from long-term observational cohorts provides additional context but cannot establish causality. The 15-year Dayton prospective cohort of 1,267 women treated with testosterone or testosterone-plus-anastrozole implants reported a 47% lower incidence of invasive breast cancer compared with SEER age-matched rates [36]. While this observation raises the possibility of a protective signal, the finding remains hypothesis-generating and requires confirmation through RCTs designed to assess oncologic outcomes. Similarly, cardiovascular safety data appear neutral, but conclusions are constrained by the absence of adequately powered trials.

In summary, the clinical application of testosterone pellets should remain restricted to carefully selected women with persistent, burdensome symptoms despite conventional therapy, and only under strict clinical monitoring. Their use must be framed as an option supported by observational data rather than high-level evidence, and clinicians should engage in shared decision-making that transparently communicates both the potential benefits and the unresolved uncertainties.

Discussion

The clinical use of subcutaneous testosterone pellets in women illustrates the persistent gap between guideline-based recommendations and real-world practice. Most international societies restrict testosterone therapy to postmenopausal women with HSDD and favor transdermal formulations [4,32]. However, the BMS [5] acknowledges that testosterone may be considered in women with persistent symptoms unrelieved by conventional therapies and notes that subcutaneous implants (pellets) are occasionally used in UK practice as part of individualized strategies. Such acknowledgment underscores the ongoing clinical demand for alternatives beyond approved formulations.

The so-called “pharmacokinetic paradox” remains a central challenge. While LC-MS/MS-based reference ranges for postmenopausal women generally place the upper total testosterone limit around 50-55 ng/dL, symptomatic benefit has been reported at higher concentrations, with therapeutic windows often extending into ranges considered supraphysiologic [9]. This discrepancy highlights the tension between frameworks that prioritize biochemical safety margins and those that emphasize patient-reported outcomes. Importantly, supraphysiologic peaks are common with pellets, even at lower doses, due to their pharmacokinetic profile. These features raise unresolved questions about efficacy, safety, and regulatory acceptance.

Safety signals have been explored across multiple domains, but interpretation is limited by reliance on observational cohorts, lack of blinding, and conflicts of interest in some registries. Reports of lower-than-expected breast cancer incidence in long-term pellet users [9,38] and retrospective registries [7,36] should be considered hypothesis-generating only, as no causal inference can be made. Similarly, cardiovascular outcomes have not demonstrated consistent excess risk in observational cohorts [11,27], and indirect reassurance from transgender men [30,31] must be interpreted with caution, given physiologic and treatment differences. Data on endometrial outcomes remain sparse, with no large biopsy-verified cohorts. Mild androgenic effects such as acne and hirsutism are common, and while usually reversible, they may affect adherence and quality of life. Psychiatric manifestations, including mood destabilization, have been reported in case studies [12], underscoring the importance of careful counseling and monitoring. Across all domains, evidence gaps persist for metabolic, lipid, and hematologic outcomes, where systematic reporting is limited.

Another layer of complexity arises from the compounded nature of most pellets. Variability in manufacturing, dosing, and insertion technique contributes to inconsistent release kinetics and undermines reproducibility. Unlike approved transdermal formulations, pellets cannot be rapidly discontinued in the event of adverse reactions, which magnifies potential safety concerns. The absence of standardized monitoring protocols further reduces comparability across studies.

Limitations of Evidence

The evidence base for testosterone pellets in women is constrained by several important methodological limitations. Most data originate from a small number of research groups and practice-based registries, often with declared or potential conflicts of interest. Study designs are predominantly non-randomized, with modest sample sizes, short follow-up periods, and reliance on self-reported outcomes. Adjustment for confounding variables such as BMI, smoking, family history, or concurrent hormone therapy is often incomplete, increasing the risk of selection bias and misclassification. The inclusion of compounded formulations further limits reproducibility, as variability in dose and manufacturing introduces heterogeneity that complicates safety interpretation. Finally, the predominance of observational designs precludes causal inference, meaning that all reported safety and efficacy signals should be interpreted as exploratory and hypothesis-generating rather than confirmatory.

Future Directions

Future research must address these gaps. Pragmatic, multicenter RCTs comparing pellets with transdermal formulations are needed, with standardized dosing, validated outcome measures, and long-term follow-up. Core outcomes should include sexual function (FSFI and FSDS-R), menopause-related quality of life (MRS), androgenic adverse events, hematocrit, lipid profiles, liver enzymes, and cardiovascular and oncologic endpoints at 12, 24, and 52 weeks. Standardization of assays (preferably LC-MS/MS) and reporting of reference ranges are essential to clarify physiologic versus supraphysiologic exposures. Ethical and practical challenges remain in designing such trials, but alignment with regulatory frameworks will be crucial to support broader clinical adoption.

Until such data are available, testosterone pellets should be regarded as an off-label therapy with exploratory evidence. Observed benefits and safety signals must be interpreted as hypothesis-generating, not confirmatory. No causal inference can be made, and their use should be limited to carefully selected women within shared decision-making frameworks, under structured monitoring of serum testosterone, hematocrit, and clinical adverse effects.

## Conclusions

Subcutaneous testosterone pellets remain a therapeutic option of interest in women’s health due to their sustained-release profile and long dosing intervals. Observational studies and long-term clinical experience suggest potential benefits in domains such as sexual function, psychological well-being, vitality, body composition, and bone health. However, these signals derive largely from uncontrolled or practice-based cohorts, many with conflicts of interest, heterogeneous assays, and non-standardized dosing. Current safety data are likewise observational and should be interpreted cautiously. Cohorts that reported lower-than-expected rates of invasive breast cancer or neutral cardiovascular outcomes did so without randomized comparators, with limited person-years of follow-up, and variable case ascertainment. These associations are hypothesis-generating and insufficient to infer protection or equivalence to approved formulations. In the absence of adequately powered randomized trials, testosterone pellets should be reserved for carefully selected peri- and postmenopausal women with persistent symptoms unresponsive to approved therapies, and only within a framework of shared decision-making and structured monitoring (e.g., LC-MS/MS testosterone levels, hematocrit, liver enzymes, and androgenic adverse effects).

Future research should prioritize pragmatic randomized trials with standardized pellet composition, LC-MS/MS-based monitoring, clearly defined primary endpoints (e.g., FSFI, FSDS-R, and MRS), and long-term safety evaluation (androgenic events, hematologic impact, lipids, liver function, blood pressure, and metabolic outcomes). Until further evidence emerges, use should be individualized and supported by structured follow-up.
